# Drug Release of Hybrid Materials Containing Fe(II)Citrate Synthesized by Sol-Gel Technique

**DOI:** 10.3390/ma11112270

**Published:** 2018-11-14

**Authors:** Michelina Catauro, Elisabetta Tranquillo, Federico Barrino, Ignazio Blanco, Francesco Dal Poggetto, Daniele Naviglio

**Affiliations:** 1 Department of Engineering, University of Campania “Luigi Vanvitelli”, via Roma 29, I-81031 Aversa, Italy; elisabetta.tranquillo@unicampania.it (E.T.); federicobarrino92@hotmail.it (F.B.); 2 Department of Civil Engineering and Architecture and UdR-Catania Consorzio INSTM, University of Catania, Viale Andrea Doria 6, 95125 Catania, Italy; iblanco@unict.it; 3 Ecoricerche srl, via Principi Normanni 36, 81043 Capua Caserta, Italy; ecoricerchesrl@virgilio.it; 4 Department of Chemical Sciences, University of Naples Federico II, via Cintia, 80126 Naples, Italy; naviglio@unina.it

**Keywords:** sol-gel, ferrous citrate, FTIR analysis, drug release, antibacterial properties

## Abstract

The use of oral iron integration is commonly recommended for the treatment of iron deficiency, nevertheless the diagnosis and treatment of this disease could clearly be improved. The aim of this work was the synthesis of therapeutic systems, iron (II) based, by sol-gel method. In an SiO_2_ matrix, we embedded different weight percentages of polyethylene glycol (PEG_6, 12, 24 wt%_) and ferrous citrate (Fe(II)C_5, 10, 15 wt%_) for drug delivery applications. Fourier Transform Infrared (FTIR) spectroscopy was used to study the interactions among different components in the hybrid materials. Release kinetics in a simulated body fluid (SBF) were investigated and the amount of Fe^2+^ released was detected by Ultraviolet–Visible spectroscopy (UV-VIS) after reaction with ortho-phenantroline. Furthermore, the biological characterization was carried out. The bioactivity of the synthesized hybrid materials was evaluated by the formation of a layer of hydroxyapatite on the surface of samples soaked in SBF using FTIR spectroscopy. Finally, also, the potential antibacterial properties of the different materials against two different bacteria, *E. coli* and *P. aeruginosa*, were investigated.

## 1. Introduction

Iron is very important for many biological functions such as respiration, energy production, DNA synthesis, and cell proliferation [1]. Several ways have been observed by the human body to conserve iron; in fact, it is strongly regulated by different molecular mechanisms, avoiding its accumulation to a toxic level or its deficiency [2]. Iron deficiency is considered the main cause of anemia worldwide, [3] and it is a major health problem that is related to maternal and child mortality [4]. The use of oral iron supplementation for the treatment of iron deficiency is usually recommended, such as ferrous sulphate, anhydrous ferrous sulphate ferrous gluconate, and ferrous fumarate [4], nevertheless, the diagnosis and treatment of this disease could clearly be improved. In fact, although oral iron supplementation is the treatment of choice for the majority of patients because of its effectiveness and safety, oral iron supplementation has disadvantages, including poor compliance, high incidence of adverse gastrointestinal effects and high potential for interactions with other treatments [5,6]. In the last decade, the production of new biological systems able to release iron (II) in a controlled manner are an interesting alternative compared to systemic therapy.

Therefore, the aim of this work was the synthesis of iron (II)-based drug delivery systems, by sol-gel method. In an SiO_2_ matrix were embedded different weight percentages of polyethylene glycol (PEG_6, 12, 24 wt%_) and ferrous citrate (Fe(II)C_5, 10, 15 wt%_) to obtain several hybrid materials. The advantages of the use of these materials as biomedical implants, for example as prosthesis or subcutaneous implants for local delivery in the systemic circulation of Fe^2+^, are the continuous release of Fe^2+^, without the need to remember to take the drug daily, also, it is possible always active coverage, because the molecule could dissolve slowly, giving a delayed effect over time. Furthermore, the use of drug delivery system optimizes the biopharmaceutical, pharmacokinetic and pharmacodynamics properties of a drug, reducing side-effects [6] 

The sol-gel method is an interesting technique to prepare organic-inorganic hybrid materials which involves the hydrolysis reactions of the precursor leading to the formation of a colloidal suspension and its evolution in a gel by condensation process [7]. The main advantages of the sol-gel techniques for the preparation of hybrid materials are the low temperatures used during the synthesis and versatility [8]. Judenstein and Sanchez have proposed the classification of the hybrid materials which depends of the interactions between the components. In particular, in the Class I the organic and inorganic compounds are bond by hydrogen bonds, van der Waals, or ionic bonds; in the Class II, the two components are linked by strong chemical bonds (covalent or polar covalent bonds) [9].

In the literature, there are many works about the silica-based materials [10,11,12], that describe the high biological properties, the cell-material interactions and cell invasion due to their reaction products [10]. The biocompatibility of silica materials has been improved with the addition of polyethylene glycol (PEG), because the presence of the polymer improving cell adhesion and growth for the high hydrophilicity of the materials [13,14,15]. PEG is used in several biomedical applications such as drug delivery thanks to his versatility and biocompatibility [16]. Moreover, the addition of PEG in the hybrids improve the reduction of toxicity and the extension of the circulation time of many drug nanocarriers [17,18,19]. 

Furthermore, in silica/PEG materials has been added ferrous citrate (Fe(II)C), prepared by the redox reaction between iron powder and citric acid, to evaluate the Fe^2+^ release from different materials and, thus, the use of hybrids as systems for controlled iron release. 

Fourier Transform Infrared (FTIR) spectroscopy was used to study the interactions among different components in the hybrid materials. Release kinetics in a simulated body fluid (SBF) were investigated and the amount of Fe^2+^ released was detected by UV–VIS spectroscopy after reaction with ortho-phenantroline. In addition, the biological characterization was carried out. The bioactivity of the different hybrids was evaluated by observing the typical peaks of hydroxyapatite on the surface of materials, soaked in SBF, using FTIR spectroscopy. Finally, also, the potential antibacterial properties of the different materials against two different bacteria, *E. coli* and *P. aeruginosa*, were investigated.

## 2. Materials and Methods

### 2.1. Ferrous Citrate Preparation

A total of 25 g Citric acid monohydrate (>99.5%, Fluka, Munich, Germany) were completely dissolved in 500 mL of ultrapure water to which 6.0 g of iron powder (>99%, Sigma-Aldrich, St. Louis, MO, USA) were added. The solution was boiled and stirred under magnetic stirring (Sigma-Aldrich, St. Louis, MO, USA) until all iron powder disappeared and consequently appeared the ferrous citrate complex (Fe(II)C). Then the mixture was cooled at room temperature and the precipitate grey/pearly complex was filtered under vacuum with a paper filter and washed with water; finally, the obtained solid was freeze dried.

### 2.2. Sol-Gel Synthesis of the Hybrid Materials

The hybrid materials with different percentages of polyethylene glycol (PEG, MW = 400, Sigma-Aldrich, St. Louis, MO, USA) and Fe(II)C were synthesized by sol-gel method. Tetraethyl orthosilicate (TEOS; Si(OC_2_H_5_)_4_; Sigma-Aldrich, St. Louis, MO, USA) was used as precursor of silica matrix and it was added in a solution of 99.8% ethanol (EtOH, Sigma-Aldrich, St. Louis, MO, USA) and distilled water. In this study the molar ratios of solution were equal to H_2_O/TEOS = 26.6 and EtOH/TEOS = 6.

**Figure 1:**
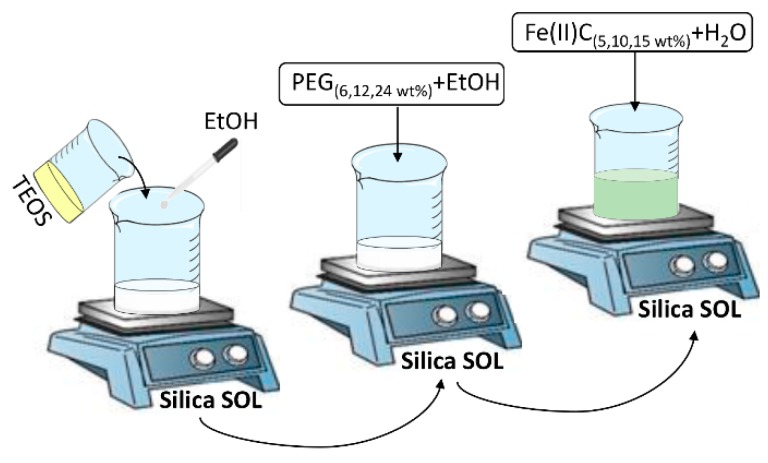
Flow chart of sol-gel synthesis.

Afterwards, different amount of PEG_(6, 12, 24 wt%)_ and Fe(II)C_(5, 10, 15 wt%)_ were solubilized in 99.8% ethanol and distilled water under stirring at 38 °C, respectively. Afterwards to the gelation process, the residual solvent was removed from the wet gels in an oven at 50 °C for 24 h. The Figure 1 shows the flow chart of the hybrid synthesis.

### 2.3. Fourier Transform Infrared (FTIR) Analysis of the Hybrid Materials

A Prestige 21 Shimadzu (Kyoto, Japan) FTIR instrument equipped with a DTGS detector was used to study the interactions between the different components. In the 400–4000 cm^−1^ region, with resolution of 4 cm^−1^ (45 scans) were recorded the spectra of the materials by Fourier transform infrared (FTIR) transmittance. KBr pelletized disks containing 2 mg of each sample and 198 mg of KBr were made. The FTIR spectra were processed by Prestige software (IR solution, software version 1.50, Shimadzu, Kyoto, Japan)

### 2.4. Study of In Vitro Release

The hybrid materials were crushed to a powder using an agate mortar (Sigma-Aldrich, St. Louis, MO, USA) and were soaked in 10 mL of simulated body fluid (SBF) solution under continuous magnetic stirring at 150 rpm.

The study of in vitro release is used to evaluated the amount of Fe^2+^ released from the materials in function of the percentages of Fe(II)C in the hybrids.

In particular, the release of Fe^2+^ from different materials was assessed at different exposure time using normative reference APAT IRSA CNR Man. 29/2003 Metodo 3160/A. In this regard, 1mL of SBF solution with the powder was added to a solution of water, hydroxylamine hydrochloride 10%, 1,10-phenanthroline 0.1%, Acetate Sodium 10%.

Release measurements were taken by means of a Shimadzu UV-1700 Double Beam Scanning UV-VIS Spectrophotometer (Kyoto, Japan). Absorbance values were taken at a wavelength λ = 520.0 nm, corresponding to the absorbance of Fe^2+^. The release experiments were repeated three times on each hybrid. Data are presented as means ± standard deviation for a representative experiment.

The calibration curve was determined by taking absorbance versus concentration between 0 and 4.99 mg/L as parameters. For this interval, the calibration curve fits the Lambert and Beers’ law [20]

A = ε l C = 1.265 C,(1)

where A is absorbance, ε is molar absorptivity coefficient, l is path length and C is concentration (mg/L).

### 2.5. Bioactivity Test

The simulated body fluid (SBF) solution was used for 7, 14, 21 days at 37 °C to study the hybrids’ bioactivity [21]. The materials powders were obtained using a mortar. The FTIR and XRD analysis were used to evaluate the apatite formation. XRD analysis was carried out using a Philips 139 diffractometer (Amsterdam, The Netherlands) equipped with a PW 1830 generator, tungsten lamp and Cu anode, where the source 140 of X-ray is given by a Cu-Kα radiation (λ = 0.15418 nm). The nucleation of biominerals on the samples could allow the depletion of the ionic species in the SBF, to overcome this problem the solution was replaced every 2 days. The ratio between the total exposed surface and the volume solution was chosen in agreement with the literature. After the exposure, the samples powders were dried in a glass desiccator (Sigma-Aldrich, St. Louis, MO, USA), and then subjected to FTIR analysis.”

### 2.6. Antibacterial Activity

In order to study the effect of the samples on the microbial growth, *E. coli* (ATCC 25922) and *P. aeruginosa* (ATCC 10145) were used. *E. coli* was cultured in TBX Medium (Tryptone Bile X-Gluc) (Liofilchem, Roseto degli Abruzzi, Italy), while *P. aeruginosa* in Pseudomonas CN Agar (Liofilchem, Roseto degli Abruzzi, Italy).

Afterwards, the bacterial cultures were diluted in distilled water to produce a bacterial cell suspension of 10 × 10^5^ CFU/mL. Both bacteria were inoculated in absence and in presence of the hybrid materials. *E. coli* was incubated with the materials for 24 h at 44 °C, while the *P. aeruginosa* for 48 h at 36 °C. The microbial growth was evaluated by observing the diameter of the inhibition halo (ID). The obtained values are the mean standard (SD) deviation of measurements carried out on samples analyzed three times.

## 3. Results

### 3.1. FTIR Analysis

**Figure 2:**
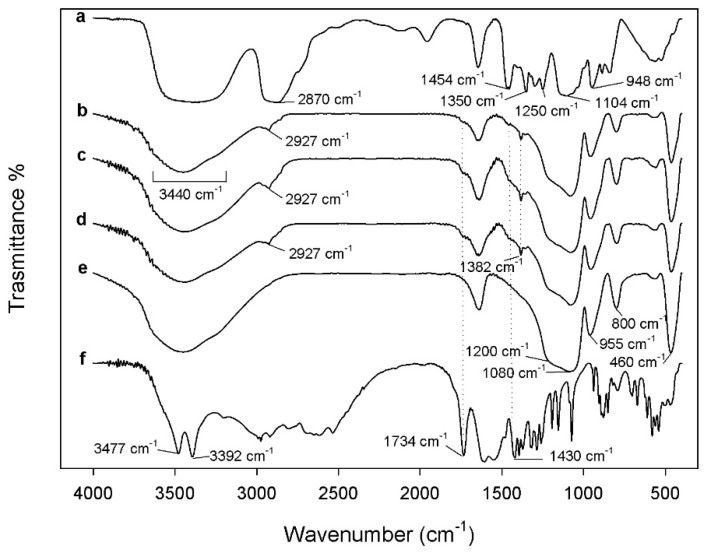
Representative FTIR spectra of (**a**) pure PEG; (**b**) SiO_2_/PEG_12 wt%/_Fe(II)C_5 wt%_; (**c**)SiO_2_/PEG_12 wt%/_Fe(II)C_10 wt%_; (**d**) SiO_2_/PEG_12 wt%_/Fe(II)C_15 wt%_; (**e**) pure SiO_2_; (**f**) pure Fe(II)C.

FTIR analysis is considered as an appropriate method to study the interactions in the hybrid materials. The typical bands of silica matrix and polymer are visible in the hybrids’ spectra which are shown in the Figure 2 (curve b–d). The asymmetric and symmetric Si–O–Si stretching, and bending vibrations of the silica are attributed to the band at 1080 cm^−1^ with a shoulder at 1200 cm^−1^ and the peaks at 800 cm^−1^ and 460 cm^−1^, respectively [22,23]. Furthermore, in the hybrid spectra, it is possible to observe a slight shift toward lower wavenumbers of the Si–OH bond vibrations and O–H stretching compared to pure SiO_2_ (curve e) from 960 to 955 cm^−1^ and from 3450 to 3440 cm^−1^ [24,25], suggesting that an interaction between silica matrix, PEG and Fe(II)C occurred. 

These interactions can be confirmed, also, by the shift of wavenumbers of some polymer peaks, in the hybrids’ spectra, the PEG methylene C–H asymmetric stretching at 2927 cm^−1^ and the band of CH_2_ group at 1382 cm^−1^ are observed, while, the same peaks, in the pure PEG spectra (curve a) at 2870 and 1350 cm^−1^ are recorded [26].

The presence of Fe(II)C is confirmed comparing the hybrid spectra with those of the pure Fe(II)C. When the different amounts of Fe(II)C are added in the SiO_2_/PEG/Fe(II)C some bands related to citrate are detectable with several intensity. In particular, the peaks at 1734 and 1430 cm^−1^ due to the C=O stretching and COO^−^ group, respectively, are visible as shoulder in the hybrid spectra (curve b–d) compared to pure Fe(II)C spectra (curve f) [27,28].

Therefore, the formation of hydrogen bonds between the –OH groups of the silica matrix and ethereal oxygen atoms (H-bond donors) or terminal –OH in the polymer chains [25,29], and, also, the interaction with Fe(II)C were suggested by the different shape and position of the silica bands.

### 3.2. Study of In Vitro Release

**Figure 3:**
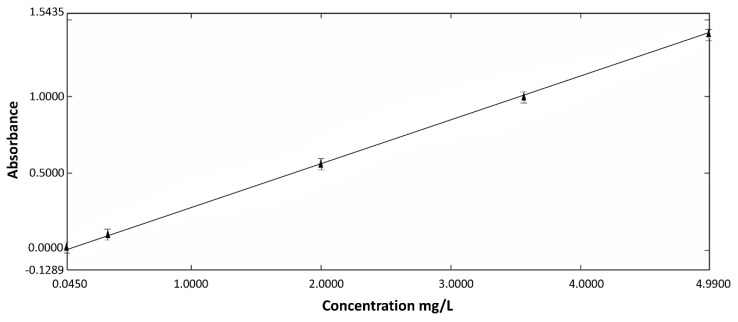
Calibration curve (520.0 nm) based on the concentration of Fe^2+^ (r^2^ = 0.9973).

Controlled Fe^2+^ release from hybrid materials is an interesting alternative to systemic therapy. The study of drug release is required to understand treatment continuity and its efficacy. The release was assayed by measuring the UV absorbance at λ = 520.0 nm related to the concentration of the solution. The calibration curve was drawn for a standard solution with different concentrations from 0 to 4.99 mg/L (Figure 3).

**Figure 4:**
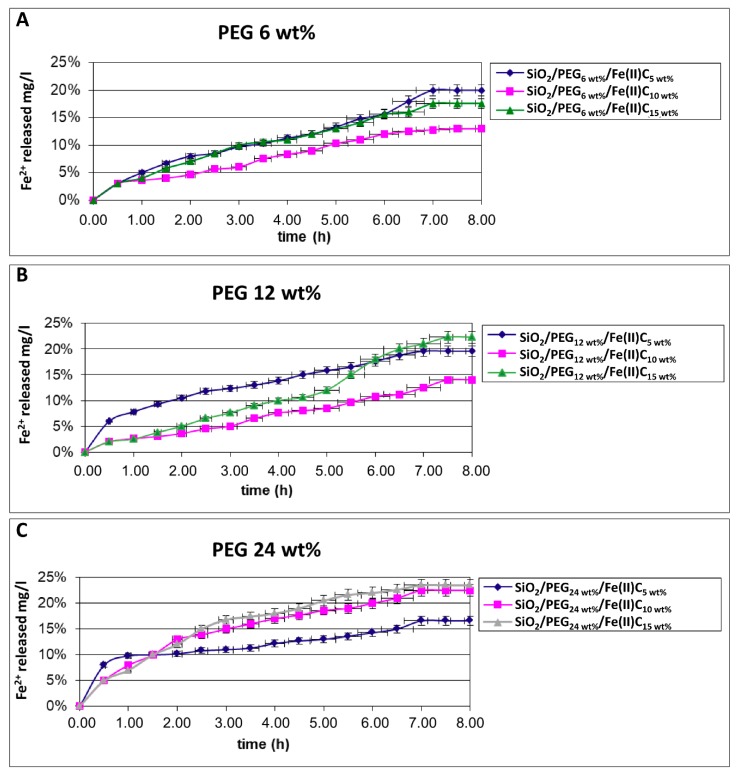
Time-dependent drug release plot for (**A**) SiO_2_/PEG_6 wt%_/Fe(II)C_5 wt%_; SiO_2_/PEG_6 wt%_/Fe(II)C_15 wt%_; SiO_2_/PEG_6 wt%_/Fe(II)C_20 wt%_; (**B**) SiO_2_/PEG_12 wt%_/Fe(II)C_5 wt%_; SiO_2_/PEG_12 wt%_/Fe(II)C_10 wt%_; SiO_2_/PEG_12 wt%_/Fe(II)C_15 wt%_; (**C**) SiO_2_/PEG_24 wt%_/Fe(II)C_5 wt%_; SiO_2_/PEG_24 wt%_/Fe(II)C_10 wt%_; SiO_2_/PEG_24 wt%_/Fe(II)C_15 wt%_. Data are presented as means ± SD for a representative experiment.

The several amount of the polymer in SiO_2_/Fe(II)C hybrids modulates the drug release differently. Comparing the different release plots (Figure 4A–C), it is possible to observed that the release of Fe^2+^ is completed within 8 h without any evident difference in the time of release. Initially, the drug release is due to dissolution and diffusion of the Fe^2+^ incorporated near the surface of the samples, afterwards, the release depends on the different type of interaction between Fe(II)C and PEG and silica.

A similar behavior was observed in the SiO_2_/PEG_(6, 12, 24 wt%)_/Fe(II)C_(5 wt%)_ hybrids (Figure 4A–C), despite the different amounts of polymer. Probably, the release of Fe^2+^ do not depend of polymer concentration but it might be due to a low percentage of Fe(II)C in the hybrid that allows many interactions with silica-PEG matrix, reducing the release of Fe^2+^.

In the release plots (Figure 4A–C) of SiO_2_/PEG_(6, 12, 24 wt%)_/Fe(II)C_(10 wt%)_ and SiO_2_/PEG_(6, 12, 24 wt%)_/Fe(II)C_(15 wt%)_ the presence of the different percentages of polymer modified the Fe^2+^ release curves, unlike the hybrids with Fe(II)C_(5 wt%)_. The PEG amount equal to 24 wt% (Figure 4C) determines a greater release of the Fe^2+^ compared to another one, probably due to the formation of the silica/PEG network. In particular, when the content of PEG and Fe(II)C is higher in the hybrids, a part of it cannot form H-bonds because all hydroxyl groups of silica are already involved in H-bonds. Probably, the formation of few weak bonds between Fe(II) and silica-PEG matrix allow the greater release of Fe^2+^ [30,31,32].

### 3.3. Bioactivity Test

**Figure 5:**
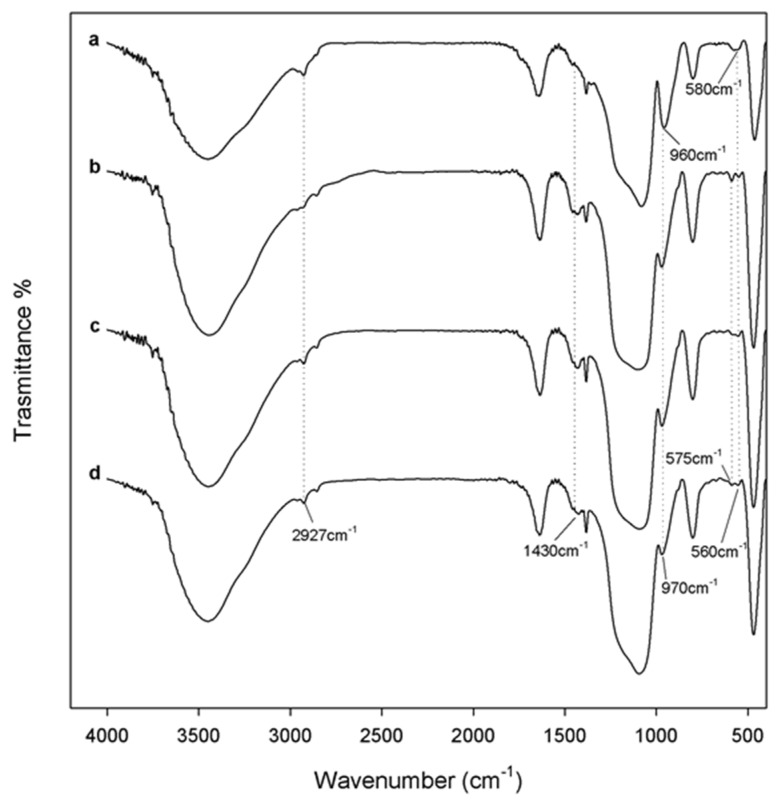
Representative FTIR spectra of (**a**) SiO_2_/PEG_12 wt%/_Fe(II)C_5 wt%_ not soaked in SBF; (**b**) SiO_2_/PEG_12 wt%/_Fe(II)C_5 wt%_; (**c**) SiO_2_/PEG_12 wt%/_Fe(II)C_10 wt%_; (**d**) SiO_2_/PEG_12 wt%/_Fe(II)C_15 wt%_; after 21 days of exposure to SBF.

The bioactive materials are able to form an apatite layer on their surfaces in the living body, allowing the bond to living bone. The presence of various functional groups on materials surface induce the hydroxyapatite formation. In this regard, the hybrids were soaked in SBF for 7, 14 and 21 days to evaluate their bioactivity [21]. In particular, the interaction of Si–OH groups on the hybrids’ surface with the Ca^2+^ ions present in the fluid allow to the hydroxyapatite nucleation and leading to an increase of positive surface charge. The formation of amorphous phosphate is induced by the combination of the Ca^2+^ ions and negative charges of the phosphate ions, which spontaneously transforms into hydroxyapatite [Ca_10_(PO_4_)_6_(OH)_2_] [33]. The FTIR was used to identify the typical peaks of hydroxyapatite layer on materials surface (Figure 5). All hybrid materials showed the same results independently of PEG and Fe(II)C content. The displacement of Si–OH band, from 960 to 970 cm^−1^ suggested the interaction of the hydroxyapatite layer with the –OH groups of the silica matrix. Furthermore, comparing the hybrid materials spectra (Figure 5 curve) after 21 days in SBF with those of the same materials not soaked in SBF (Figure 5 curve a), the split of the band at 580 cm^−1^ in two new ones at 575 and 560 cm^−1^, was visible. The presence of these new bands is caused by the formation of the hydroxyapatite precipitate, in particular, and are due to the stretching of the hydroxyl groups of hydroxyapatite and the vibrations of the P$O_{4}^{3-}$ groups [34,35]. Moreover, the peak related to citrate at 1430 cm^−1^ attributed to COO^−^ group, undergoes change in the intensity that might be due to the interaction with the hydroxyapatite layer.

**Figure 6:**
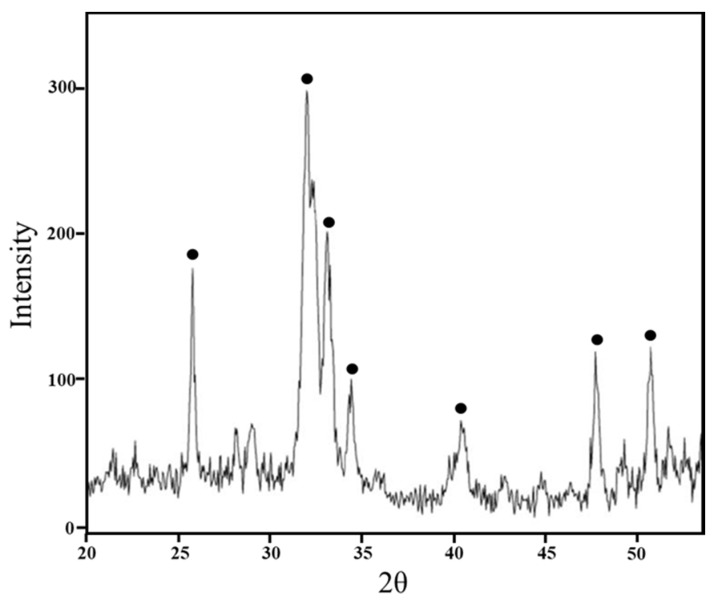
A representative XRD of SiO_2_/PEG_12 wt%_/Fe(II)C_15 wt%_ soaked in SBF solution for 21 days.

Furthermore, XRD measurement was used to identify the presence of hydroxyapatite on the hybrids’ surface. The intense peaks of hydroxyapatite crystalline are visible in Figure 6(**●**), confirming that the materials surface is covered by hydroxyapatite layer after 21 days in SBF. Moreover, this result suggest that the hydroxyapatite layer is very thick to detect XRD signals from hybrids’ surface [21,36].

### 3.4. Antibacterial Activity

**Figure 7:**
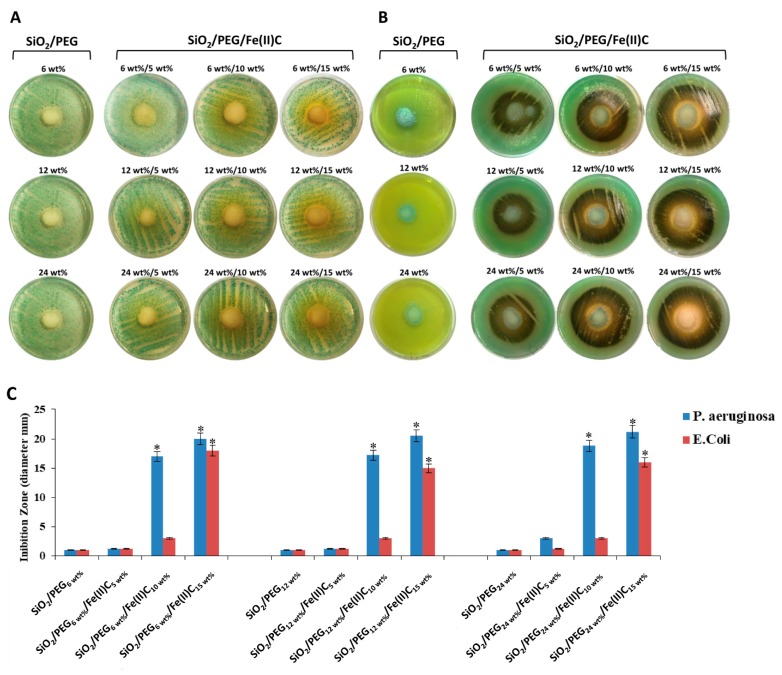
(**A**) Inhibition halo (ID) of *E. coli* with all materials; (**B**) Inhibition halo (ID) of *P. aeruginosa* with all materials; (**C**) Comparison of inhibition zone of *E. coli* and *P. aeruginosa* for all materials. Values are the mean SD of measurements carried out on samples analyzed three times. The means and S.D. are shown. *, *p* <0.05 vs the bacteria control treated with hybrids without Fe(II)C or vs the bacteria treated with hybrids containing Fe(II)C.

In order to evaluate the effect of the hybrids on the microbial growth, *E. coli* and *P. aeruginosa* were used. The different materials were grinded in a mortar to obtain powders. Both bacteria were inoculated in absence and in presence of the 100 mg of hybrids’ powders. The zones of inhibition formed by the materials without Fe(II)C and containing different amount of Fe(II)C against *E. coli* are shown in Figure 7A. It is possible to observed that the materials exhibited mild activity against *E. coli*, only the SiO_2_/PEG_6 wt%_/Fe(II)C_15 wt%_ hybrid induces the inhibition halo around the synthesized hybrid Figure 7C. These results suggest that the microbial growth was inhibited by higher amount of Fe(II)C and by low percentage of PEG. When the *P. aeruginosa* was inoculated in presence of all the hybrids, the inhibition halo was more visible (Figure 7B). In particular, the diameter of zone of inhibition (Figure 7C) increases with 10 and 15 wt% of Fe(II)C, independently of the polymer content. Furthermore, the pigmentation reddish brown of *P. aeruginosa* inoculated with the hybrids containing Fe(II)C is observed (Figure 7B). In literature is reported that *P. aeruginosa* is able to produce a range of pigments under certain growth conditions [37,38,39]. Probably, the pigmentation reddish brown it is due to the production of pyomelanin which is involved in iron reduction and acquisition [40], resistance to oxidative stress [41] and virulence [38]. The pyomelanin could be produced by the release of Fe^2+^ from hybrids as this phenomenon is not observed with materials without Fe(II)C. Finally, in the Figure 7C are reported the inhibition zones of *E. coli* and *P. aeruginosa*. Comparing the diameter of the inhibition zones of the different hybrids, the better antibacterial activity against *P. aeruginosa* was observed. 

## 4. Conclusions

In the present study, the different percentage of Fe(II)C and PEG were added in the silica matrix by sol-gel method. FTIR spectra of the obtained hybrids, show all bands of citrate, and also, the typical peaks of polymer and silica. Therefore, the different shape and position of some bands confirm the formation of H-bonds between the inorganic matrix, the PEG chains and, also, the interaction with Fe(II)C. The release of Fe^2+^ from hybrids was studied. Comparing all materials, those containing 24 wt% of PEG determine a greater release of the Fe^2+^. This result might be due to the high content of PEG and Fe(II)C, that doesn’t allow a part of it to form H-bonds because all hydroxyl groups of silica are already involved in weak bonds. Furthermore, the FTIR analysis after 21 days in SBF has suggest that the hybrids are bioactive, and the presence of Fe(II)C or polymer does not affect the bioactivity of silica matrix. Finally, *E. coli* and *P. aeruginosa* were inoculated with the different hybrid materials to evaluate their antibacterial activity. The materials exhibited mild activity against *E. coli* compared to *P. aeruginosa*. In particular the diameter of zone of inhibition of *P. aeruginosa* increases with 10 and 15 wt% of Fe(II)C, independently of polymer content. In conclusion the obtained hybrid materials could be used as biomedical implants to release different controlled amount of Fe^2+^ in the systemic circulation, without going through the gastrointestinal track, in order to allow active coverage. Obviously, these hybrid materials will be investigated further to confirm and extend the obtained results.
